# Sleep Quality and Pain Empathy Among Nursing Interns: The Serial Mediating Roles of Burnout and Moral Identity

**DOI:** 10.1155/jonm/1480675

**Published:** 2026-07-30

**Authors:** Xiangqi Xu, Hui Jiang, Zhenghui Zhang, Yushang Li, Baojian Wei

**Affiliations:** ^1^ School of Nursing, Shandong First Medical University & Shandong Academy of Medical Sciences, Jinan 250000, China, sdfmu.edu.cn; ^2^ Department of Basic Medicine, Heze Medical College, Heze 274000, China

**Keywords:** burnout, moral identity, nursing interns, pain empathy, sleep quality

## Abstract

**Background:**

Empathy for patients’ pain is a core component of compassionate nursing care and is increasingly recognised as important for care quality and workforce sustainability. Nursing interns commonly experience poor sleep quality and burnout during clinical placement, yet the interrelationships between these factors and pain empathy remain unclear.

**Aim:**

To examine the association between sleep quality and pain empathy among nursing interns and the potential serial mediating roles of burnout and moral identity in this association.

**Design:**

A multicentre cross‐sectional study.

**Methods:**

A convenience sample of 1108 final‐year nursing interns undertaking clinical placements at 102 public hospitals in East and North China was recruited between October and November 2025 through participating nursing schools. Data were collected using the Pittsburgh Sleep Quality Index, Maslach Burnout Inventory, Moral Identity Measure and Empathy for Pain Scale. Descriptive statistics and Pearson correlations were performed using SPSS 27.0. Serial mediation was examined using PROCESS Macro (Model 6) with 5000 bootstrap resamples after adjustment for age, gender and educational level.

**Results:**

Poorer sleep quality, higher burnout and lower moral identity were each significantly associated with lower pain empathy (all *p* < 0.001). Burnout and moral identity demonstrated significant independent and serial indirect associations in the relationship between sleep quality and pain empathy, with the total indirect effect accounting for 31.43% of the total effect.

**Conclusions:**

Sleep quality was significantly associated with pain empathy among nursing interns, with burnout and moral identity demonstrating significant independent and serial indirect associations. Strategies targeting sleep health, burnout and professional value development may be relevant for supporting empathic care during the transition to professional nursing practice.

**Implications for Nursing Management:**

Nurse managers should consider sleep‐supportive placement scheduling, early burnout monitoring and value‐based mentorship strategies to strengthen empathy, wellbeing and workforce readiness among nursing interns.

## 1. Introduction

Empathy for patients’ pain is widely regarded as a core component of compassionate nursing care and contributes to improved care quality, higher patient satisfaction and stronger therapeutic relationships [1]. Pain is one of the most common and distressing symptoms encountered in healthcare [2], and responsive pain care reflects the core values of humanistic nursing, including compassion, dignity and responsiveness to patient needs [3]. In practice, pain empathy is not simply an affective reaction to another person’s suffering [4]; rather, it underpins the recognition, interpretation and timely, patient‐centred response to pain [5]. This process requires not only affective responsiveness but also the cognitive appraisal of another person’s experience [6]. Broader research on emotion and empathy similarly suggests that understanding others’ experiences involves interactions between affective responses and cognitive appraisal [7, 8].

Although related to general empathy, pain empathy is conceptually distinct. General empathy refers to the capacity to understand and share others’ emotional experiences, whereas pain empathy specifically concerns recognising, interpreting and responding to another person’s pain. Given the central role of pain assessment and management in nursing care, pain empathy may be particularly relevant for clinical practice and patient outcomes. For nursing interns, who represent a crucial pipeline for the future nursing workforce, the development of pain empathy during clinical training is therefore especially important for supporting patient‐centred care and preparing an empathic and sustainable future nursing workforce [9].

Supporting empathy development among nursing interns has become more pressing in the context of the global nursing shortage and concerns about workforce sustainability [10]. Early clinical experiences are pivotal in shaping nursing students’ professional identity and have been associated with longer‐term commitment to and retention in the nursing profession [11]. When empathy is weak, patient experience may suffer, but interns may also become increasingly frustrated, detached and disconnected to the meaning of nursing work [12]. For nursing management and clinical education, understanding the factors associated with pain empathy during internship is therefore of practical importance for developing supportive educational environments and promoting workforce wellbeing during the transition to professional practice [11]. Emerging emotion research further suggests that emotional functioning is dynamic and context‐dependent, evolving through continuous interactions between individuals and their environments [13, 14].

Clinical placement is a demanding transition period that requires effective educational and organisational support. Nursing interns are expected to adapt to rotating schedules, workload pressures, unfamiliar routines and the emotional burden of direct patient care [15]. These challenges may compromise sleep quality and increase vulnerability to burnout [16]. Sleep quality is a multidimensional indicator of sleep health, encompassing sleep continuity, efficiency, restoration and daytime functioning [17]. Burnout, typically characterised by emotional exhaustion, depersonalisation and reduced personal accomplishment, reflects progressive depletion of emotional and occupational resources [18, 19]. Although poor sleep quality and burnout have each been linked to lower empathy, their combined relationship with pain empathy remains insufficiently understood despite their potential implications for intern wellbeing, clinical performance and workforce sustainability [20, 21].

Moral identity may represent a key explanatory factor in these associations. Moral identity refers to the extent to which moral values are internalised within the self‐concept and may strengthen prosocial motivation, ethical sensitivity and caring behaviour [22, 23]. Although related to professional identity, moral identity specifically focuses on the internalisation of moral values rather than identification with the nursing role or profession. It is also conceptually distinct from moral resilience, which refers to the capacity to maintain ethical functioning under adversity. Unlike compassion fatigue, which reflects strain arising from repeated exposure to suffering, moral identity represents a value‐based psychological resource that may support compassionate care. This perspective may be particularly relevant for nursing interns, whose professional values are still developing during clinical training [24].

Moreover, little is known about how these factors operate simultaneously within a theoretically integrated framework during clinical placement. Existing studies have primarily focused on registered nurses or have examined only isolated bivariate associations, with limited attention to the interrelationships among sleep quality, burnout, moral identity and pain empathy [21]. Research examining these interrelationships among nursing interns remains particularly limited, despite internship representing a critical period for professional socialisation, value formation and transition to clinical practice.

Clarifying these interrelationships is relevant to nursing management because generic support strategies may be insufficient when the underlying connections among these factors are poorly understood [25]. Such understanding may help inform more targeted strategies to support intern wellbeing and empathy during clinical training. Evidence regarding the associations among sleep quality, burnout and moral identity may help inform internship scheduling, supervisory support, mentorship arrangements and early burnout prevention strategies during clinical placement [16]. Therefore, this study examined the association between sleep quality and pain empathy among nursing interns and the potential serial mediating roles of burnout and moral identity. Specifically, we tested a serial mediation model to provide evidence that may inform educational and organisational strategies to strengthen empathy, wellbeing and workforce readiness among future nurses.

## 2. Theoretical Framework and Hypotheses

This study was grounded primarily in Conservation of Resources (COR) theory and supplemented by Psychological Capital perspectives and Social Cognitive Theory, as each framework explains a distinct component of the proposed associations. Specifically, COR theory explains how poor sleep quality may initiate resource depletion and increase vulnerability to burnout, Psychological Capital perspectives help explain how depleted resources may undermine value‐based internal resources such as moral identity, and Social Cognitive Theory explains how moral identity may shape empathic behaviour.

**FIGURE 1:**
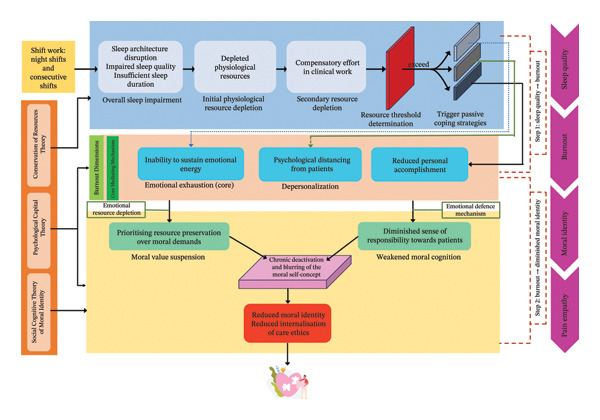
Proposed serial mediation model of sleep quality and pain empathy.

We therefore proposed a serial mediation model in which the association between sleep quality and pain empathy involves burnout and moral identity in sequence (see Figure 1).

### 2.1. Theoretical Integration and Hypothesis Development

COR theory suggests that individuals strive to obtain, preserve and protect valued resources, whereas resource loss generates stress and increases vulnerability to further depletion [26]. In the internship context, sleep can be understood as a foundational physiological resource that supports emotional regulation, cognitive functioning and adaptive performance [16]. Poor sleep quality may therefore place nursing interns at greater risk of burnout during clinical training [27].

Burnout can be conceptualised as progressive depletion of psychological and occupational resources [25]. For nursing interns, persistent exhaustion and disengagement may diminish emotional availability and responsiveness to patients, thereby compromising empathic functioning [28, 29]. From a COR perspective, burnout may also diminish the cognitive and emotional energy needed for value‐based self‐regulation. Consequently, caring values may become less salient in daily practice, making moral identity more difficult to sustain under pressure.

Psychological Capital perspectives emphasise that internal psychological resources can buffer stress and promote adaptive functioning [30]. Although Psychological Capital was not examined as a study variable, this perspective provides a useful framework for understanding how resource depletion may undermine later value‐based functioning. In the present study, moral identity was conceptualised as a value‐based internal resource reflecting the extent to which moral and professional values are integrated within the self‐concept [23, 24]. When burnout accumulates, interns may have fewer emotional and cognitive resources available to maintain value‐based self‐regulation, providing a theoretical basis for hypothesising that burnout precedes moral identity in the proposed pathway.

Social Cognitive Theory further explains how moral identity may shape behaviour [31]. When caring values such as relieving suffering, respecting dignity and responding to patient needs become central to self‐definition, individuals may be more likely to respond empathically [23, 32]. In nursing practice, this may be reflected in stronger pain empathy [22]. Thus, moral identity may function as an internal regulatory mechanism that translates professional values into empathic behaviour, particularly during the transition to professional nursing practice and under demanding clinical conditions.

Taken together, poor sleep quality may initiate a process of resource loss that increases burnout [16, 25]. Burnout may in turn reduce the salience of moral values through emotional exhaustion and disengagement, whereas lower moral identity may lessen attentiveness and responsiveness to patients’ pain experiences [22, 23]. Accordingly, sleep quality may be associated with pain empathy through both direct associations and a hypothesised serial pathway involving burnout and moral identity.

### 2.2. Research Hypotheses

**FIGURE 2:**
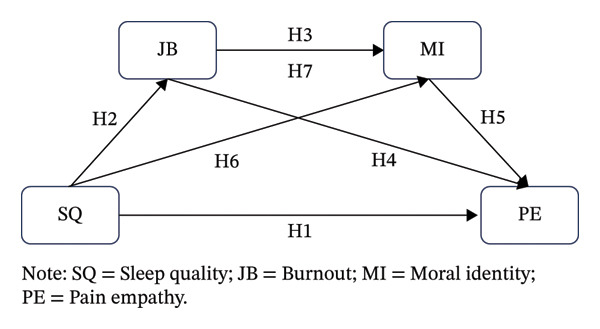
Hypothesised serial mediation model.

Accordingly, the following hypotheses were proposed (see Figure 2):

 H1. Poorer sleep quality is associated with lower pain empathy.
 H2. Poorer sleep quality is associated with higher burnout.
 H3. Higher burnout is associated with lower moral identity.
 H4. Higher burnout is associated with lower pain empathy.
 H5. Higher moral identity is associated with higher pain empathy.
 H6. Moral identity mediates the association between sleep quality and pain empathy.
 H7. Burnout and moral identity sequentially mediate the association between sleep quality and pain empathy.

## 3. Participants and Methods

### 3.1. Study Design

A multicentre cross‐sectional study was conducted to examine the associations among sleep quality, burnout, moral identity and pain empathy among nursing interns. A serial mediation model was used to test the hypothesised pathways.

### 3.2. Participants

Between October and November 2025, participants were recruited through convenience sampling during supervised clinical placements at 102 public hospitals in East and North China, with recruitment coordinated through participating nursing schools. In this study, nursing interns were defined as final‐year nursing students undertaking supervised full‐time clinical placements as the final stage of preregistration nursing education. The study population comprised final‐year nursing students undertaking supervised full‐time clinical placements at public hospitals in East and North China during the study period. Participating hospitals were predominantly tertiary hospitals, with several secondary hospitals also represented.

Participants were eligible if they (1) were final‐year nursing students, (2) had undertaken clinical placement for at least 3 months and (3) provided informed consent. Participants were excluded if they (1) were placed in units without direct patient care responsibilities, such as administrative departments or central sterile supply units, (2) had completed less than 3 months of placement because of leave or other interruptions, (3) had experienced recent major physical or life events likely to substantially affect physical or psychological status (e.g. surgery, fractures, bereavement or serious accidents) or (4) had severe physical or mental conditions that could interfere with questionnaire completion.

### 3.3. Sample Size Estimation

Sample size was estimated using G∗Power 3.1. Assuming a medium effect size (*f* ^2^ = 0.15) [33], a significance level of *α* = 0.05, statistical power of 0.80 and three predictors, the minimum required sample size was 129. A total of 1108 participants were included in the final analysis, substantially exceeding the minimum requirement and ensuring adequate statistical power. Age, gender and educational level were included as covariates based on theoretical relevance, previous literature and potential confounding effects.

### 3.4. Ethical Considerations

Ethical approval was obtained from the Ethics Committee of Shandong First Medical University (Approval No. 202604240361). The study was conducted in accordance with the Declaration of Helsinki [34]. Before participation, participants were informed of the study purpose, procedures, voluntary nature of participation, confidentiality safeguards and their right to withdraw at any time without penalty. Written informed consent was obtained prior to questionnaire completion. Participation or nonparticipation did not affect students’ clinical placement arrangements, academic evaluation or academic rights and interests. Relevant clinical education departments were informed to support study implementation during placement training.

### 3.5. Instruments

#### 3.5.1. General Information Questionnaire

A researcher‐developed questionnaire was used to collect demographic, placement‐related, individual and contextual variables that could potentially confound the associations of interest. The questionnaire included demographic characteristics (4 items), internship characteristics (4 items), individual traits and experiences (7 items) and internship contextual support (1 item).

#### 3.5.2. Pittsburgh Sleep Quality Index (PSQI)

Sleep quality was measured using the Chinese version of the PSQI [35, 36]. The PSQI comprises seven components, each scored from 0 to 3, yielding a global score ranging from 0 to 21, with higher scores indicating poorer sleep quality. A global score > 7 indicates poor sleep quality. Cronbach’s *α* was 0.924 in the present study. Permission to use the PSQI was obtained via email prior to data collection.

#### 3.5.3. Maslach Burnout Inventory (MBI)

Burnout was measured using the Chinese version of the MBI [18, 37]. The 22‐item instrument comprises three dimensions: emotional exhaustion (9 items), depersonalisation (5 items) and reduced personal accomplishment (8 items). Responses are rated on a seven‐point Likert scale ranging from 0 to 6, with higher scores indicating greater burnout. Cronbach’s *α* was 0.966 in the present study.

#### 3.5.4. Moral Identity Measure (MIM)

Moral identity was assessed using the Chinese version of the MIM [23, 38]. The 10‐item scale comprises two dimensions, internalisation and symbolisation, each containing five items. Responses are rated on a five‐point Likert scale, yielding a total score ranging from 10 to 50, with higher scores indicating stronger moral identity. Cronbach’s *α* was 0.922 in the present study.

#### 3.5.5. Empathy for Pain Scale (EPS)

Pain empathy was measured using the Chinese version of the EPS [39, 40]. The instrument comprises 12 items administered across four pain‐related scenarios and includes two dimensions: psychophysical discomfort response (9 items) and empathic response (3 items). Item scores were calculated as the mean across the four scenarios, yielding a total score ranging from 12 to 60, with higher scores indicating stronger pain empathy. Cronbach’s *α* was 0.871 in the present study.

### 3.6. Data Collection

Paper‐based questionnaires were administered between October and November 2025 after permission had been obtained from the relevant teaching administration departments of participating schools and hospitals. Standardised procedures were followed across all sites. A total of 1213 questionnaires were returned, of which 1108 were retained for analysis after excluding 105 invalid responses, yielding a valid response rate of 91.3%.

All measures were self‐reported. To minimise common method bias, anonymous completion, a balanced mix of positively and negatively worded items and an intermixed presentation of items from different scales were used during data collection. To preserve participant anonymity, information identifying individual placement hospitals was not collected. Consequently, all observations were analysed at the individual level, and potential between‐hospital variation could not be formally modelled.

### 3.7. Statistical Analysis

Data were analysed using SPSS 27.0 and PROCESS Macro Version 5.0. Common method bias was examined using Harman’s single‐factor test. The first unrotated factor accounted for 16.83% of the total variance, indicating no substantial common method bias. Descriptive statistics and Pearson’s correlation analyses were performed for all study variables.

Serial mediation was tested using PROCESS Model 6, with burnout and moral identity specified as mediators. Age, gender and educational level were entered as a priori covariates. Variance inflation factors indicated no multicollinearity (all VIFs < 5). Indirect effects were estimated using 5000 bias‐corrected bootstrap resamples. Effects were considered statistically significant when the 95% confidence interval excluded zero. All tests were two‐tailed, with *α* set at 0.05.

## 4. Results

### 4.1. Participant Characteristics

A total of 1108 nursing interns were included in the final analysis. Participants had a mean age of 20.69 years (SD = 0.61). Of the sample, 55.2% were female (*n* = 612) and 44.8% were male (*n* = 496). Participants were predominantly enrolled in diploma‐level nursing programmes (59.5%, *n* = 659), whereas 40.5% (*n* = 449) were undergraduate nursing students. More than half of the participants were undertaking clinical placement in tertiary hospitals (53.7%, *n* = 595), and 66.3% (*n* = 735) had completed less than 6 months of placement. Further participant characteristics are presented in Table 1.

**TABLE 1:** Participant characteristics.

Variable	Category	Frequency	Percentage (%)	Cumulative percentage (%)
Gender	Male	496	44.77	44.77
	Female	612	55.23	100.00

Age	19.0	8	0.72	0.72
	20.0	421	38.00	38.72
	21.0	627	56.59	95.31
	22.0	48	4.33	99.64
	23.0	4	0.36	100.00

Educational level	Diploma	659	59.48	59.48
	Undergraduate	449	40.52	100.00

Clinical placement hospital level	Grade III‐A	595	53.70	53.70
	Grade III‐B	226	20.40	74.10
	Grade II‐A	281	25.36	99.46
	Grade II‐B	6	0.54	100.00

Current duration of clinical placement	Within 3 months	342	30.87	30.87
	Within 6 months	735	66.34	97.20
	Within 9 months	29	2.62	99.82
	Within 12 months	2	0.18	100.00

Attitude towards nursing	Very positive	123	11.10	11.10
	Positive	264	23.83	34.93
	Neutral	369	33.30	68.23
	Negative	244	22.02	90.25
	Very negative	108	9.75	100.00

Per capita monthly family income	≤ RMB 3000	183	16.52	16.52
	RMB 3001–6000	430	38.81	55.32
	RMB 6001–10000	255	23.01	78.34
	RMB 10001–15000	198	17.87	96.21
	> RMB 15000	42	3.79	100.00

Prior outreach/volunteer experience	Yes	559	50.45	50.45
	No	549	49.55	100.00

Personality type	Extrovert	356	32.13	32.13
	Ambivert	356	32.13	64.26
	Introvert	396	35.74	100.00

Prior hospitalisation/caregiver experience	Yes	498	44.95	44.95
	No	610	55.05	100.00

Previous experience of major trauma or severe pain	Yes	267	24.10	24.10
	No	841	75.90	100.00

Current or past chronic pain (duration ≥ 3 months)	Yes	392	35.38	35.38
	No	716	64.62	100.00

Self‐rated pain tolerance	Very low	132	11.91	11.91
	Low	331	29.87	41.79
	Moderate	350	31.59	73.38
	High	225	20.31	93.68
	Very high	70	6.32	100.00

Prior training in pain empathy care	Yes	472	42.60	42.60
	No	636	57.40	100.00

Empathy competence of clinical instructors	Very high	168	15.16	15.16
	High	333	30.05	45.22
	Moderate	311	28.07	73.29
	Low	270	24.37	97.65
	Very low	26	2.35	100.00

Total		1108	100.00	100.00

### 4.2. Descriptive Statistics of Sleep Quality, Burnout, Moral Identity and Pain Empathy Among Nursing Interns

Descriptive statistics for sleep quality, burnout, moral identity and pain empathy are presented in Table 2. Among the subdimensions, emotional exhaustion and habitual sleep efficiency showed the highest mean scores within burnout and sleep quality, respectively.

**TABLE 2:** Descriptive statistics of the core variables and their dimensions (*n* = 1108).

Construct and dimension	Observed range	Mean ± SD
Pittsburgh Sleep Quality Index (total)	0.00∼21.00	10.11 ± 5.56
Habitual sleep efficiency	0.00∼3.00	1.60 ± 1.11
Sleep latency	0.00∼3.00	1.42 ± 0.95
Sleep disturbances	0.00∼3.00	1.43 ± 0.95
Subjective sleep quality	0.00∼3.00	1.40 ± 0.92
Sleep duration	0.00∼3.00	1.44 ± 0.91
Use of sleep medication	0.00∼3.00	1.38 ± 0.91
Daytime dysfunction	0.00∼3.00	1.44 ± 0.94
Maslach Burnout Inventory (total)	40∼137.00	88.36 ± 25.74
Emotional exhaustion	12.00∼63.00	40.30 ± 12.32
Depersonalisation	7.00∼35.00	20.09 ± 5.98
Reduced personal accomplishment	11.00∼53.00	31.92 ± 9.40
Moral Identity Measure (total)	10.00∼47.00	28.73 ± 8.65
Internalisation	5.00∼25.00	14.51 ± 4.79
Symbolisation	5.00∼25.00	14.21 ± 4.17
Empathy for Pain Scale (total)	18.25∼48.50	33.28 ± 4.84
Empathic response	4.25∼12.00	8.27 ± 1.26
Psychophysical discomfort response	14.00∼36.50	25.01 ± 3.74

### 4.3. Correlation Analysis of Sleep Quality, Burnout, Moral Identity and Pain Empathy

**FIGURE 3:**
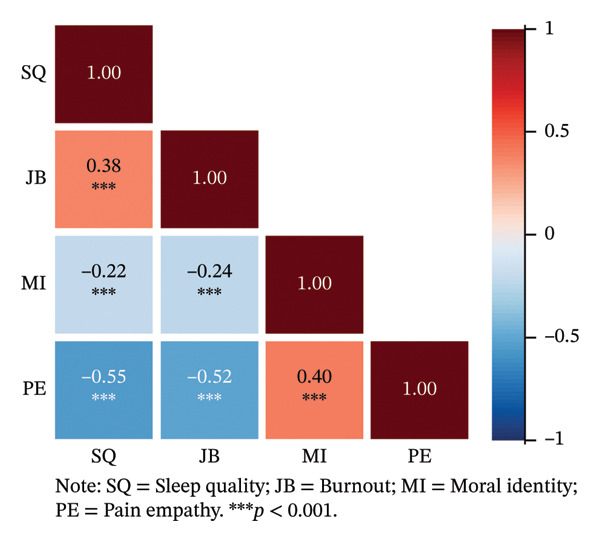
Heatmap of Pearson correlations among the study variables.

Pearson’s correlation analyses indicated significant correlations among sleep quality, burnout, moral identity and pain empathy (see Figure 3). Poorer sleep quality was associated with higher burnout and lower moral identity and pain empathy. Burnout was negatively associated with both moral identity and pain empathy, whereas moral identity was positively associated with pain empathy.

### 4.4. Regression Analysis of Pain Empathy Among Nursing Interns

A multiple linear regression analysis was conducted with pain empathy as the dependent variable and sleep quality, burnout and moral identity as predictors. After controlling for demographic variables, the overall model was significant (*F* (3, 1104) = 325.67, *p* < 0.001), accounting for 46.8% of the variance in pain empathy (adjusted *R* ^2^ = 0.468). Sleep quality and burnout were negatively associated with pain empathy, whereas moral identity was positively associated with pain empathy. No multicollinearity was detected (see Table 3).

**TABLE 3:** Linear regression analysis.

	Unstandardised coefficients		Standardised coefficient	*t*	*p*	Collinearity diagnostics	
	*B*	SE	Beta			VIF	Tolerance
Constant	3.172	0.050	—	63.142	< 0.001	—	—
Sleep quality	−0.192	0.012	−0.378	−15.778	< 0.001	1.193	0.838
Moral identity	0.112	0.011	0.239	10.496	< 0.001	1.082	0.924
Burnout	−0.110	0.008	−0.319	−13.284	< 0.001	1.203	0.831

*Note:* Dependent variable = pain empathy. *R* ^2^ = 0.469, adjusted *R* ^2^ = 0.468, *F* (3, 1104) = 325.671.

### 4.5. Serial Mediation Analysis of the Relationship Between Sleep Quality and Pain Empathy

PROCESS Model 6 with 5000 bootstrap resamples was used to examine the serial mediating roles of burnout and moral identity in the relationship between sleep quality and pain empathy, controlling for age, gender and educational level.

As given in Table 4, poorer sleep quality was significantly associated with higher burnout, lower moral identity and lower pain empathy. Burnout was negatively associated with both moral identity and pain empathy, whereas moral identity was positively associated with pain empathy in the final model. These findings were consistent with H1–H5.

**TABLE 4:** Serial mediation analysis of the relationship between sleep quality and pain empathy.

Analysis section	Path	*B*	SE	Statistic	*p*	95% CI
Burnout model	SQ ⟶ JB	0.558	0.041	13.631	< 0.001	[0.478, 0.639]

Model fit	*R* ^2^ = 0.144, adjusted *R* ^2^ = 0.143, *F* (1, 1106) = 185.808, *p* < 0.001					

Moral identity model	SQ ⟶ MI	−0.165	0.034	−4.857	< 0.001	[−0.232, −0.099]
	JB ⟶ MI	−0.133	0.023	−5.738	< 0.001	[−0.178, −0.087]

Model fit	*R* ^2^ = 0.076, adjusted *R* ^2^ = 0.074, *F* (2, 1105) = 45.352, *p* < 0.001					

Total effect model	SQ ⟶ PE	−0.280	0.013	−21.988	< 0.001	[−0.305, −0.255]

Model fit	*R* ^2^ = 0.304, adjusted *R* ^2^ = 0.304, *F* (1, 1106) = 483.466, *p* < 0.001					

Direct effect model	SQ ⟶ PE	−0.192	0.012	−15.778	< 0.001	[−0.216, −0.168]
	JB ⟶ PE	−0.110	0.008	−13.284	< 0.001	[−0.126, −0.094]
	MI ⟶ PE	0.112	0.011	10.496	< 0.001	[0.091, 0.132]

Model fit	*R* ^2^ = 0.469, adjusted *R* ^2^ = 0.468, *F* (3, 1104) = 325.671,*p* < 0.001					

Indirect effects	SQ ⟶ JB ⟶ PE	−0.061	0.011+	−5.810	< 0.001	[−0.083, −0.039]
	SQ ⟶ MI ⟶ PE	−0.018	0.008+	−2.300	0.021	[−0.034, −0.002]
	SQ ⟶ JB ⟶ MI ⟶ PE	−0.008	0.003+	−2.395	0.017	[−0.014, −0.002]

Total indirect effect (SQ ⟶ PE)		−0.088	0.014+	−6.113	< 0.001	[−0.115, −0.061]

Total effect (SQ ⟶ PE)		−0.280	0.013	−21.988	< 0.001	[−0.305, −0.255]

*Note:* The 95% confidence intervals for indirect effects were estimated using the normal approximation based on the reported standard errors. JB = burnout; + denotes bootstrap standard error.

Abbreviations: MI = moral identity, PE = pain empathy, SQ = sleep quality.

The total effect of sleep quality on pain empathy was significant (*B* = −0.280, 95% CI [−0.305, −0.255]). After burnout and moral identity were entered into the model, the direct effect remained significant (*B* = −0.192, 95% CI [−0.216, −0.168]), accounting for 68.57% of the total effect. The total indirect effect was significant (*B* = −0.088, 95% CI [−0.115, −0.061]), accounting for 31.43% of the total effect.

**FIGURE 4:**
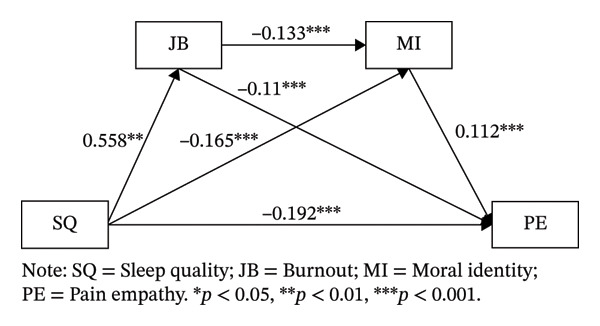
Standardised path coefficients for the serial mediation model.

Three significant indirect pathways were identified: through burnout alone, through moral identity alone and through burnout and moral identity sequentially. The independent indirect pathways via burnout and moral identity together accounted for 28.22% of the total effect, with the pathway through burnout showing the largest effect size. These findings were consistent with the hypothesised indirect pathways in H6 and H7. The final serial mediation model is shown in Figure 4.

## 5. Discussion

This multicentre study examined the relationship between sleep quality and pain empathy among nursing interns and further explored the potential mediating roles of burnout and moral identity. Three principal findings emerged. First, poorer sleep quality was significantly associated with lower pain empathy. Second, burnout and moral identity each showed significant independent indirect associations in this relationship. Third, a significant hypothesised serial pathway involving sleep quality, burnout, moral identity and pain empathy was supported. Collectively, the indirect associations accounted for 31.43% of the total association. Taken together, these findings suggest that pain empathy during internship may be associated with immediate psychological states as well as broader factors related to physiological recovery, occupational strain and professional value internalisation. Although the proportion of male participants was higher than commonly reported among nursing students, gender was included as an a priori covariate in all adjusted analyses. Accordingly, the observed associations were interpreted after accounting for differences in gender composition.

More broadly, the findings indicate that empathy in clinical training may be better understood as a dynamic professional capacity rather than a fixed personal trait [9]. During internship, empathic functioning is likely to fluctuate in response to workload, fatigue, learning pressure and the quality of supervisory support [9, 41]. This perspective is particularly relevant to nursing management, because reductions in empathy during training may have implications for patient‐centred care, workforce wellbeing and longer‐term professional development and workforce retention [9, 10].

### 5.1. Sleep Quality and Pain Empathy Among Nursing Interns

The present study found that poorer sleep quality was associated with lower pain empathy, suggesting that inadequate recovery may be associated with lower empathic functioning during internship. This is consistent with evidence that poor sleep quality may reduce emotional sensitivity, interpersonal responsiveness and prosocial behaviour [42]. The internship period may be particularly vulnerable because nursing interns commonly face rotating schedules, unfamiliar clinical environments, frequent evaluation and ongoing academic and clinical demands [15]. Under these combined pressures, inadequate sleep may compromise the psychological resources required for empathic care [42].

Pain empathy requires the ability to recognise pain‐related verbal and nonverbal cues, regulate one’s own emotional responses and adopt the patient’s perspective [43, 44]. These processes depend on attention, working memory and executive control, all of which may be weakened by poor sleep quality [45]. In clinical settings, this may not appear as overt indifference, but rather as delayed responses, reduced communicative warmth, diminished sensitivity to subtle distress signals [46] or a greater reliance on task‐focused interactions when workload intensifies [47, 48].

This finding is clinically meaningful because pain empathy is closely linked to accurate pain assessment, patient trust, treatment adherence and perceived quality of care [49]. For interns, repeated patterns of reduced empathic engagement may gradually become normalised and may shape future professional habits and attitudes towards patient care [41]. Protecting sleep health during placement may therefore benefit not only nursing interns’ wellbeing, but also patient‐centred care, workforce readiness and the quality of future nursing care [16, 42].

### 5.2. The Mediating Role of Burnout

Burnout showed a significant independent indirect association in the relationship between sleep quality and pain empathy. This finding is plausible because insufficient recovery sleep and occupational burnout often coexist and may reinforce one another over time in healthcare settings [50]. When restorative sleep is limited, emotional fatigue may accumulate more rapidly in the face of clinical workload demands, increasing vulnerability to exhaustion, cynicism and disengagement [51, 52].

Burnout may be particularly consequential during internship because nursing interns often have limited control over schedules and workflow while simultaneously facing substantial emotional demands [15, 50]. They are expected to master technical skills, adapt to workplace norms and respond professionally to patient suffering while still developing coping strategies [53]. Under such circumstances, emotional exhaustion may reduce the capacity for empathic engagement, whereas depersonalisation may promote emotional distancing as a defensive response [51].

These findings suggest that the association between poor sleep quality and empathy is not merely a matter of tiredness or reduced energy [42]. Rather, this association may reflect broader occupational strain and difficulties in sustaining empathic engagement during internship [41, 51]. From a management perspective, early monitoring and support for burnout during training may be beneficial rather than waiting until workforce entry [28]. Supportive supervision, psychologically safe learning climates, manageable workloads and structured debriefing following emotionally demanding placements may help preserve empathic capacity during professional socialisation [53].

### 5.3. The Mediating Role of Moral Identity

Moral identity also showed a significant independent indirect association in the relationship between sleep quality and pain empathy. This extends previous nursing research by suggesting that empathy may be associated not only with transient emotional states, but also with the extent to which caring values are integrated into the self‐concept [54].

For nursing interns, moral identity may function as an internal professional compass that sustains patient‐centred behaviour when external demands are high. Individuals who strongly identify with values such as compassion, dignity and responsibility may be more motivated to notice suffering, respond respectfully and remain engaged in the relational aspects of care even when tired or stressed. Conversely, under persistent strain, professional ideals may become less salient, and immediate task demands may increasingly dominate behavioural priorities [31, 55].

This finding is important because moral identity remains relatively underexplored in workforce preparation research despite its relevance to professional formation [55]. Internship may represent a sensitive developmental period during which values are translated from classroom ideals into workplace behaviour. Role modelling by senior nurses, ward culture, mentor feedback and opportunities for reflective learning may all strengthen or weaken this process [53]. Empathy development may therefore be enhanced when communication skills training is combined with strategies that actively cultivate professional values.

### 5.4. The Serial Pathway of Sleep Quality, Burnout, Moral Identity and Pain Empathy

A key contribution of this study was the support for a significant hypothesised serial pathway linking sleep quality, burnout, moral identity and pain empathy. Although the serial indirect association was modest in magnitude, it remained statistically significant, suggesting that sleep quality, burnout, moral identity and pain empathy may be interrelated rather than operating as isolated factors.

From a resource perspective, insufficient sleep may contribute to impaired physiological recovery and reduced emotional regulation capacity. This resource loss may in turn contribute to higher levels of burnout [16]. Under conditions of exhaustion and detachment, individuals may have fewer emotional and cognitive resources available to sustain reflection on professional values, making moral identity more difficult to maintain. Lower salience of caring values may also be associated with reduced motivation for empathic engagement [16, 31].

This interpretation offers a more nuanced account than models treating burnout and moral identity as unrelated parallel factors. It suggests that emotional exhaustion may be associated not only with lower empathy, but also with reduced salience of the value orientation that supports compassionate behaviour [56, 57]. In this sense, empathy loss during internship may reflect both depletion of personal resources and disconnection from professional meaning [58].

Even modest pathway effects may be meaningful in complex clinical systems, where empathy is shaped by multiple interacting factors. Small but repeated reductions in empathic engagement during internship may accumulate over time and have implications for confidence, professional identity, patient communication patterns and transition readiness [59]. For educators and managers, this implies that empathy cannot be fully protected through communication training alone if fatigue and burnout remain unaddressed or if clinical learning environments inadequately support intern wellbeing [19, 42].

### 5.5. Study Strengths and Theoretical Contribution

This study has several strengths. First, it used a large multicentre sample drawn from multiple hospitals, increasing the diversity of clinical learning environments represented. Second, it focused specifically on nursing interns, a group central to future workforce sustainability yet less frequently examined than registered nurses. Third, it moved beyond single‐mediator approaches by testing linked pathways involving sleep quality, burnout, moral identity and pain empathy.

Conceptually, the study advances the literature by integrating sleep health, occupational wellbeing and professional value formation within a single explanatory framework. Previous studies have often examined these factors in relative isolation. The present findings suggest that empathy among nursing interns may be better conceptualised as a multidimensional capability shaped by interacting physiological, psychological and professional processes. This integrative perspective extends current understanding of pain empathy by bringing together sleep health, occupational wellbeing and professional value formation within a single explanatory framework.

The study further suggests that COR theory, Social Cognitive perspectives and Psychological Capital approaches may provide complementary rather than competing lenses [26, 30]. Together, these perspectives help explain how depleted resources, altered self‐regulation and weakened value orientation may converge to influence empathic behaviour. This integrative perspective may provide a valuable foundation for future intervention research aimed at improving both workforce wellbeing and quality of care.

### 5.6. Practical Implications for Nursing Management and Education

The findings have direct implications for nursing management and clinical education. Because poorer sleep quality was associated with lower pain empathy through both direct and indirect pathways, support strategies should address not only sleep health itself but also the occupational and value‐based conditions that shape empathic functioning during internship [25].

At the educational level, nursing schools should strengthen preparation for clinical placement by integrating sleep health, burnout awareness and professional formation into the curriculum. Students may benefit from structured teaching on the effects of sleep loss on attention, emotion regulation and patient‐centred care [42], together with training in self‐monitoring, stress regulation and reflective practice. Communication skills training alone may be insufficient if interns become disconnected from the values that give caring work meaning [60]; therefore, reflective discussion, narrative learning, patient experience sharing and positive role modelling by experienced nurses may help strengthen the connection between moral identity and compassionate practice [61].

At the clinical level, hospitals should recognise internship scheduling as a workforce and patient‐safety issue rather than a purely administrative matter. Where feasible, placement systems should support more predictable rostering, protected recovery time and attention to cumulative fatigue. In addition, routine wellbeing assessment and early burnout monitoring should begin during training rather than after workforce entry [52]. Supervisors and preceptors can play an important role by identifying signs of distress, providing psychologically safe supervision and offering timely support before temporary strain progresses to persistent burnout or disengagement [62]. Mentorship arrangements that include regular feedback, encouragement and visible role modelling may further help interns translate professional values into daily practice.

At the individual level, nursing interns should be encouraged to strengthen their personal protective resources through sleep hygiene, reflective journaling and active engagement with learning opportunities related to pain assessment and empathic communication. Developing greater knowledge of pain care and maintaining awareness of one’s own emotional state may help interns preserve empathic capacity under pressure.

Taken together, these findings suggest that empathy during internship is not only an individual attribute but also a modifiable outcome shaped by educational preparation, clinical support and workplace design. A coordinated approach involving nursing schools, clinical institutions and nursing interns may therefore be most effective in supporting wellbeing, empathy and readiness for professional practice through coordinated efforts to promote sleep‐supportive placement scheduling, psychologically safe supervision, early burnout monitoring and value‐based mentorship [63, 64]. Such multilevel and integrative approaches are increasingly recognised as essential for addressing complex healthcare challenges [65].

### 5.7. Limitations and Future Directions

Several limitations should be considered. First, the cross‐sectional design does not permit firm conclusions regarding temporal ordering or causality. Longitudinal studies are needed to determine whether changes in sleep quality precede later changes in burnout, moral identity and empathy. Second, all variables were measured by self‐report, which may increase common method bias and social desirability effects. Future studies could incorporate objective sleep indicators, supervisor ratings, behavioural empathy measures or mixed‐method approaches to better address the context‐sensitive nature of affective assessment [66]. Third, the model focused on selected mediators and did not include other potentially relevant factors such as resilience, emotional intelligence, clinical learning climate or supervisory support. Broader ecological models may help further clarify how empathy develops during clinical placement. Fourth, participants were recruited through convenience sampling from participating nursing schools while undertaking supervised clinical placements at public hospitals in East and North China. Because deidentified information on individual placement hospitals was not collected in the anonymous survey, potential between‐hospital variation could not be formally modelled. Future studies should retain de‐identified institutional information, incorporate broader geographic sampling and examine whether the observed associations are consistent across different clinical education settings. Finally, although statistically significant, the serial indirect effect was modest, indicating that pain empathy is likely shaped by multiple factors beyond those examined here. Future research should therefore explore more comprehensive ecological models incorporating organisational, educational and individual influences on empathic functioning. Because the serial mediation model was tested using cross‐sectional data, the proposed ordering of sleep quality, burnout, moral identity and pain empathy should be interpreted as theoretically informed rather than evidence of temporal or causal relationships.

## 6. Conclusion

Poorer sleep quality was significantly associated with lower pain empathy among nursing interns. Burnout and moral identity demonstrated significant independent and serial indirect associations, suggesting that empathy may be understood in relation to physiological strain, occupational distress and professional value internalisation. These findings highlight sleep health as a potentially modifiable target for supporting empathic care during clinical training. Interventions that jointly address sleep quality, burnout prevention and moral identity development may facilitate nursing interns’ professional adaptation and contribute to a more resilient and compassionate future nursing workforce.

## Author Contributions

Xiangqi Xu: conceptualisation, methodology, data curation, formal analysis, investigation, writing–original draft and writing–review and editing.

Hui Jiang: conceptualisation, formal analysis, writing–original draft and writing–review and editing.

Zhenghui Zhang: investigation, resources and visualisation.

Yushang Li: resources and writing–review and editing.

Baojian Wei: supervision, conceptualisation, project administration, methodology supervision and final manuscript approval.

## Funding

This study was supported by the Curriculum Ideology and Politics Teaching Research Project (No. KZ2024026) and the Education and Teaching Reform Research Project (No. XM2023026).

## Disclosure

All authors have read and approved the final manuscript.

## Ethics Statement

Ethical statement for this study was obtained from Shandong First Medical University (Approval No. 202604240361).

## Conflicts of Interest

The authors declare no conflicts of interest.

## Data Availability

The data that support the findings of this study are available from the corresponding author upon reasonable request.
